# Radiological presentation of active pulmonary tuberculosis in kidney transplant recipients: a retrospective study of four cases and a review of the literature

**DOI:** 10.1590/0100-3984.2023.0124

**Published:** 2024-05-07

**Authors:** Virgilio de Araujo Oliveira, Ricardo Augusto Monteiro de Barros Almeida, Ricardo de Souza Cavalcante, Luis Gustavo Modelli de Andrade, Sergio Marrone Ribeiro

**Affiliations:** 1 Faculdade de Medicina de Botucatu, Universidade Estadual Paulista “Júlio de Mesquita Filho” (UNESP). Botucatu, SP, Brazil

**Keywords:** Tuberculosis, pulmonary, Kidney transplantation, Radiography, thoracic, Tomography, X-ray computed, Immunosuppressive agents/adverse effects, Opportunistic infections, Tuberculose pulmonar, Transplante de rim, Radiografia torácica, Tomografia computadorizada, Imunossupressores/efeitos adversos, Infecções oportunísticas

## Abstract

Although kidney transplantation is the best therapeutic option for patients with chronic kidney disease, the immunosuppression required greatly increases susceptibility to infections that are responsible for high post-transplant mortality. Pulmonary tuberculosis (TB) represents a major cause of such infections, and its early diagnosis is therefore quite important. In view of that, we researched the manifestations of active pulmonary TB in kidney transplant recipients, through chest X-ray and computed tomography (CT), as well as determining the number of cases of active pulmonary TB occurring over a 3.5-year period at our institution. We identified four cases of active pulmonary TB in kidney transplant recipients. The CT scans provided information complementary to the chest X-ray findings in all four of those cases. We compared our CT findings with those reported in the literature. We analyzed our experience in conjunction with an extensive review of the literature that was nevertheless limited because few studies have been carried out in lowand middle-income countries, where the incidence of TB is higher.

## Abstract (pt)

Apesar de o transplante renal ser a melhor opção terapêutica para pacientes com doença renal crônica, a imunodepressão decorrente desse tratamento eleva muito a suscetibilidade desses pacientes a infecções, responsáveis por altas taxas de mortalidade pós-operatórias. A tuberculose (TB) pulmonar é uma significativa causa dessas infecções, sendo muito importante o seu diagnóstico precoce. Assim, nós pesquisamos as manifestações da TB pulmonar ativa nessa população de transplantados renais por meio de radiografias simples e tomografia computadorizada (TC) do tórax, também para estabelecer o número de casos de TB pulmonar ativa em nossa instituição após levantamento de 3,5 anos. Encontramos quatro casos de TB pulmonar ativa em pacientes transplantados renais. A TC forneceu informações adicionais em relação às radiografias de tórax em 100% dos casos analisados. Comparamos os nossos achados de TC com os relatados na literatura. Somamos a experiência obtida com extensa revisão da literatura, ainda limitada nessa questão, com poucos estudos realizados em países em desenvolvimento onde a incidência de TB é maior.

## INTRODUCTION

Kidney transplantation (KTX) is currently considered the best therapy for patients with chronic kidney disease (CKD), because it improves survival and quality of life, as well as reducing treatment costs. However, long-term use of immunosuppressive drugs is required in kidney transplant recipients (KTRs). Although these drugs prevent graft rejection, they also increase the risk of infections, such as pulmonary tuberculosis (TB). The vulnerability of KTRs is evident when we analyze their history. In addition to a history of CKD and dialysis, KTRs usually have at least one severe comorbidity, such as cardiovascular disease and diabetes mellitus, and all of those factors are also associated with immunodeficiencies^(1)^.

Reduced access to diagnosis and treatment of TB has resulted in an increase in TB-related deaths. The best estimates for 2020 are 1.3 million TB-related deaths among HIV-negative individuals (up from 1.2 million in 2019) and an additional 214,000 among HIV-positive individuals (up from 209,000 in 2019), with the combined total back to the level of 2017. The reductions in annual TB incidence rates achieved in previous years have been nearly reversed. These indicators are forecast to be much worse in 2021 and 2022^(2)^. In situations in which the immune system is impaired, TB is a significant problem and the primary means of controlling it is early detection^(3)^. It is estimated that the frequency of active pulmonary TB development in patients undergoing solid organ transplantation is 20-74 times greater than in the rest of the population, the magnitude of that difference varying by the type of organ transplanted. In one study, more than 66% of active TB cases occurred in the first year after transplantation, with a mortality rate of 20-30%^(4)^. The average risk of developing TB is estimated to be approximately 37 times higher in KTRs than in the rest of the population, and that risk can be up to 43 times higher in countries with a high prevalence of TB, such as Brazil^(5)^.

In immunocompetent patients, the imaging patterns of pulmonary TB are well established^(6)^. However, for patients who are immunosuppressed because of KTX, there is a scarcity of data in the literature, and their cases are often aggregated with those of pulmonary infection by various other etiological agents^(7-9)^, or even with cases of TB in patients undergoing transplantation of other organs such as the liver, heart, and pancreas^(10,11)^. The only study identified in the literature that was designed to analyze the patterns of the onset of pulmonary TB in KTRs through tomography-specifically high-resolution computed tomography (HRCT)-was that conducted by Pereira et al.^(12)^. Those authors found peculiarities in KTRs with pulmonary TB. In fact, we identified one other such study, carried out by Wu et al.^(13)^, although those authors employed nonstandard radiological terminology.

At our institution, approximately 120 patients per year undergo KTX. However, the number of cases of active pulmonary TB among those patients is still unknown. Due to the small number of cases in the countries producing most of the scientific literature on the topic, there have been few studies on the onset of pulmonary TB among KTRs. This study intends to expand that body of data in order to identify the radiological parameters that might help diagnose and treat TB more appropriately in this vulnerable population.

Our initial objective was to establish the number of cases of active pulmonary TB in the population of KTRs treated at our university hospital. To that end, we investigated the radiological manifestations of active pulmonary TB in KTRs on X-ray and CT, comparing the two methods. A secondary objective was to determine whether or not the patterns of radiological presentation overlap with those of the general population. By combining the experience obtained in the present study with a broad review of and comparison with data in the literature, we advanced toward the latter objective.

## MATERIALS AND METHODS

We analyzed the electronic medical records of all KTRs followed at our institution over a period of 3.5 years. The inclusion criteria were being ≥ 15 years of age and having been diagnosed with active pulmonary TB within that same period. Patients who underwent KTX concurrent with the transplantation of another organ were excluded, as were those who had active coinfection with another pathogen.

The diagnosis of active pulmonary TB was based on clinical and epidemiological criteria, together with a positive smear microscopy result, with or without a positive culture of materials such as sputum, bronchoalveolar lavage fluid, and a pulmonary biopsy specimen. Positivity for TB on a polymerase chain reaction test of any of those materials, if available, was also considered a criterion for the diagnosis.

### Imaging protocol and population

Digitized chest X-rays were obtained in two views (frontal and lateral), in accordance with international standards. In one patient, we obtained only the anteroposterior view in the standing position, because of the poor clinical condition of the patient. In addition, HRCT images were obtained, in a 16-slice scanner (Activion 16; Toshiba, Tokyo, Japan), within one week of the acquisition of the X-rays. For descriptive analysis of the findings, the Fleischner Society glossary of terms was used^(14)^.

We identified 769 patients undergoing follow-up at our kidney transplant clinic during the study period. Of those, only four (0.52%) were diagnosed with active pulmonary TB. Table 1 shows the demographic, epidemiological, clinical, diagnostic, and treatment characteristics of the patients evaluated. Of the four patients evaluated, three (75%) were women and only one (25%) was White. The mean age at which active pulmonary TB was diagnosed was 56 years (range, 41-65 years).

**Table 1:** Demographic, epidemiological, clinical, diagnostic and therapeutic characteristics of patients submitted to KTX and diagnosed with active pulmonary TB.

Variable	Patient			
	1	2	3	4
Age at transplantation (years)	62	34	57	50
Age at pulmonary TB diagnosis (years)	65	41	58	53
Sex	Male	Female	Female	Female
Race	Black	Mixed	Mixed	White
Etiology of CKD	Hypertension	Undetermined	Hypertension/lithiasis	Hypertension
Type of dialysis	Hemodialysis	Hemodialysis	Hemodialysis	Peritoneal
Time on dialysis (months)	45	18	82	88
HIV/hepatitis C virus/hepatitis B surface antigen	No/No/No	No/No/No	No/No/No	No/No/No
Diabetes mellitus	No	No	No	No
Current/former smoker	No/No	No/Yes	No/Yes	No/Yes
Current/former alcoholic	No/Yes	No/No	No/No	NR/NR
Prior TB	No	No	No	No
Contact with an active TB case	No	No	No	No
Prior prophylaxis against TB	No	No	No	No
Date of KTX	3/26/2011	8/8/2009	4/12/2015	3/8/2014
Type of donor	Deceased	Deceased	Deceased	Deceased
Immunosupression induction therapy	Anti-CD25 antibody	Not reported	Anti-CD25 antibody	ATG
Immunosupression maintenance therapy	SRL+MPS+PD	FK+SRL+PD	FK+MPS+PD	FK+MPS+PD
Immunosupression intensification in the last 6 months	No	No	No	No
Days from KTX to pulmonary TB diagnosis	1,311	2,432	143	1,165
Symptoms				
Chest pain	Yes	No	No	No
Fever	Yes	Yes	Yes	No
Appetite loss	Yes	Yes	No	No
Weight loss	Yes	Yes	No	No
Sweating	Yes	Yes	No	No
Productive cough	Yes	Yes	No	Yes
Dry cough	No	No	No	Yes
Dyspnea	No	No	No	Yes
Lymph node enlargement	No	No	Yes	No
Creatine clearance at pulmonary TB diagnosis (mL/m)	46	24	68	19
Clinical basis of the diagnosis of pulmonary TB	SSM+/SC+	SSM+/SC+	BALFC+	SSM+
Signs of active TB at other sites	Peritoneum	Cecum	Lymph node(s)	No
R/l/P/E sensitivity	NA	Yes/Yes/NA/Yes	Yes/Yes/NA/Yes	NA
TB treatment	R/l/P/E	LFX/E/S	R/l/P/E	R/l/P/E
TB treatment duration (months)	12	12	12	9
Follow-up after pulmonary TB diagnosis (days)	1,243	686	930	310
Clinical cure	Yes	Yes	Yes	Yes
Complications				
Deep vein thrombosis	Yes	Yes	No	No
Anemia	No	Yes	No	No
Colonization with *M. gordonae*	Yes	No	No	No
R/l/P/E hepatotoxicity	No	Yes	No	No
Sequelae	No	No	No	No
Acute rejection	No	No	No	Yes
Graft loss	No	No	No	No
Death	No	No	No	No

ATG, anti-thymocyte globulin (antibody); SRL, sirolimus; MPS, mycophenolate sodium; PD, prednisone; FK, tacrolimus; SSM+, positive sputum smear microscopy; SC+, positive sputum culture; BALFC+, positive bronchoalveolar lavage fluid culture; NA, not available; R, rifampin; I, isoniazid; P, pyrazinamide; E, ethambutol; LFX, levofloxacin; S, streptomycin.

The most common etiology of CKD was arterial hypertension (in 75%). The predominant type of dialysis in the pre-KTX period was hemodialysis (in 75%), and the time on dialysis ranged from 18 months to 88 months. None of the patients had diabetes or presented with any infection other than TB. There were also no patients who had had TB previously, had reported contact with an active TB case, or had received prophylaxis against TB. All transplanted kidneys were from deceased donors. The maintenance immunosuppressive therapy comprised tacrolimus and mycophenolate sodium in three of the four patients, sirolimus in two, and prednisone in all four. The immunosuppression had not been intensified in the last six months prior to the diagnosis of TB in any of the patients. The time from KTX to the diagnosis of pulmonary TB ranged from 4.8 to 81 months. All the patients presented a positive smear microscopy result, either in sputum or in bronchoalveolar lavage fluid. The diagnosis was confirmed by culture in three patients, and antimicrobial susceptibility testing (available for two of those patients) showed sensitivity to rifampin, isoniazid, and ethambutol. The duration of TB treatment ranged from 9 months to 12 months. Three patients showed evidence of active TB involvement at other sites, although that could not be confirmed. All patients progressed well during the TB treatment, achieving a clinical cure, and the infection did not reactivate. Notably, deep vein thrombosis was detected during the treatment in two of the patients. One of the patients presented pulmonary colonization with *Mycobacterium gordonae* after starting the TB treatment. During the follow-up period, there were no apparent sequelae, graft losses, or deaths, although mixed acute rejection occurred in one patient.

**Figure 1:**
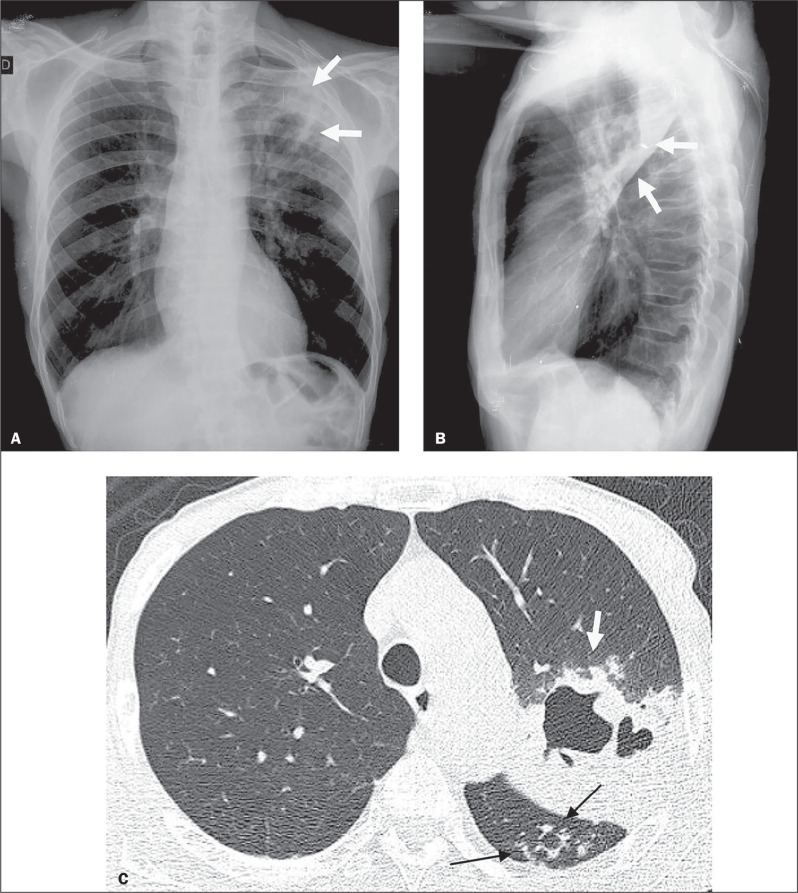
Chest X-ray (A,B) and HRCT (C) of a 66-year-old male with pulmonary TB (patient 1) showing consolidation with central cavitation (white arrows). On HRCT, a tree-in-bud pattern was seen at multiple sites, including the upper segment of the left lower lobe (black arrows).

**Figure 2:**
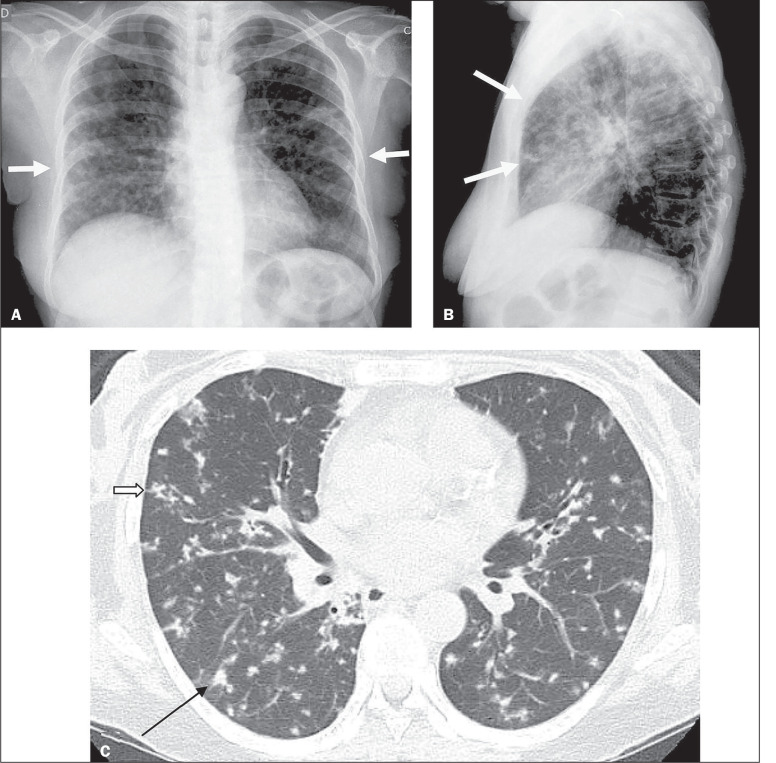
Chest X-ray (A,B) and HRCT (C) of a 41-year-old female with pulmonary TB (patient 2). A,B: Chest X-ray showing a miliary pattern (white arrows). C: HRCT showing small centrilobular nodules with branching (open arrow) and airspace nodules (black arrow).

**Figure 3:**
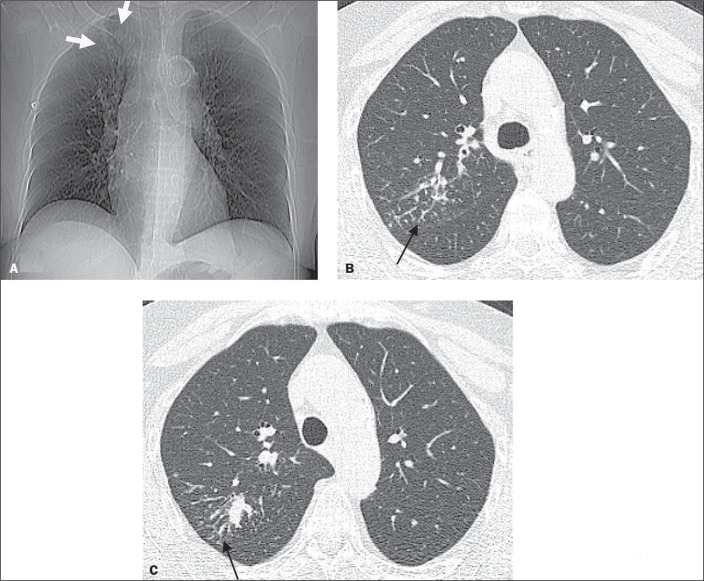
Chest X-ray (A) and HRCT (B,C) of a 58-year-old female with pulmonary TB (patient 3). A: Chest X-ray showing a reticular pattern characterized by mild interstitial opacity in the upper right lung (white arrows), better visualized on HRCT. B,C: HRCT showing a tree-in-bud pattern (black arrows).

**Figure 4:**
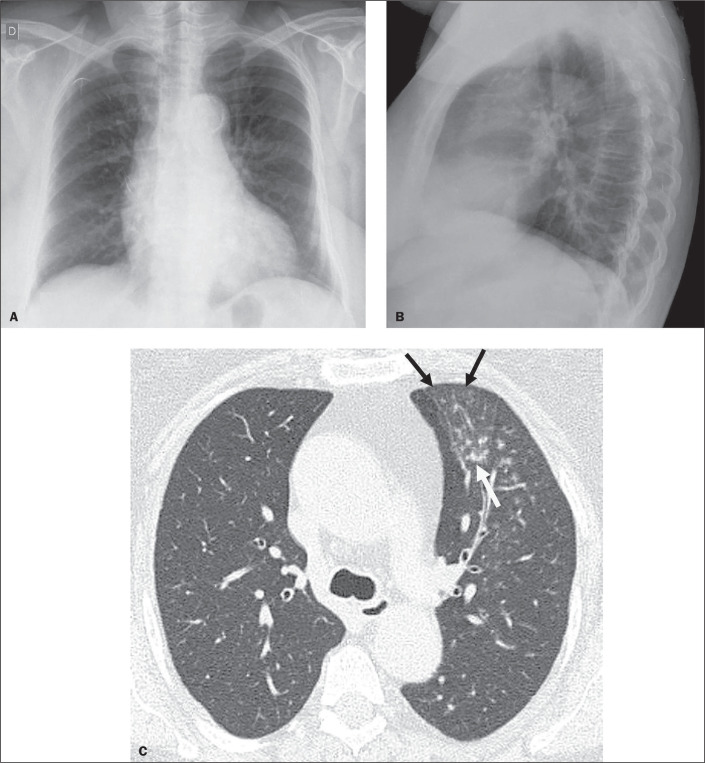
Chest X-ray (A,B) and HRCT (C) of a 53-year-old female with pulmonary TB (patient 4). A,B: Chest X-ray showing no lung abnormalities. C: HRCT showing a tree-in-bud pattern (white arrow) and ground-glass opacity (black arrows).

The imaging exams were evaluated by two radiologists, and disagreements were resolved by consensus. The radiologists evaluated chest X-ray and HRCT images of all four patients, through targeted protocols, providing a descriptive analysis of each one. Pulmonary manifestations of active pulmonary TB were assessed with HRCT, which showed recurring small centrilobular nodules with branching (tree-in-bud pattern) in all four patients. We observed consolidation in three patients, a miliary pattern in one, ground-glass opacity in one, cavitation in one, and lymph nodes with calcification in one patient (Table 2; Figures 1 to 4).

**Table 2:** Chest X-ray and HRCT findings in patients undergoing KTX and diagnosed with pulmonary TB.

Patient	Chest X-ray findings	Chest HRCT findings
1	Alveolar opacity, with central cavitation, in the left upper lobe	Consolidation, with central cavitation in the apicoposterior segment of the left upper lobe Small centrilobular nodules, some showing branching (tree-in-bud pattern) and others coalescing, together with interstitial opacities in the upper segment of the lower left lobe Five mediastinal lymph nodes, less than 1 cm in size, in subcarinal chains and the right hilum, presenting complete, dense, homogeneous calcifications
2	Diffuse reticulonodular interstitial opacity with a miliary pattern	Bilateral random/miliary distribution, together with a tree-in-bud pattern Airspace nodules, some coalescing and forming small areas of consolidation, mainly in the upper lobes
3	Poorly defined reticular pattern in the right upper lobe	Small centrilobular nodules with branching (tree-in-bud pattern), some coalescing and forming small areas of consolidation in the posterior and apical segments of the right upper lobe
4	No abnormalities	Small centrilobular nodules, with branching (a tree-in-bud pattern) in areas of ground-glass opacity, in the anterior segment of the left upper lobe and medial basal segment of the left lower lobe

## DISCUSSION AND REVIEW

In KTRs, a wide range of bacterial, mycobacterial, fungal, viral, and parasitic organisms can cause pulmonary infections, often with nonspecific signs and symptoms^(9)^. Increasingly, KTX appears to be the treatment of choice for CKD. The prevalence of TB is higher in low-and middle-income countries than in high-income countries, and the number of cases of the disease among KTRs is consequently greater in the former^(5)^.

In Brazil, despite the high overall prevalence of TB, it is evident that there is considerable socioeconomic disparity between the different regions of the country, especially when the northern region and northeastern region are collectively compared with the southern region and southeastern region (where our institution is situated), the latter two regions presenting the best health care and economic conditions. The fact that our institution is located in the southeastern region can explain the small number of cases of pulmonary TB found in our study sample, which was nonetheless within the range expected and reported the literature^(5,12)^.

Pereira et al.^(12)^ evaluated 4,128 patients undergoing KTX at two transplant centers in Brazil and identified pulmonary TB in 40 KTRs (0.96%), a proportion higher than that found in our study. Confirming the wide variability of imaging findings in this condition, the authors found that the pulmonary manifestations on CT included a miliary pattern (seen in 40%); cavitation and centrilobular nodules (in 22.5%); ground-glass opacity and consolidation (in 15%); lymph node enlargement (in 12.5%); and pleural effusion (in 10%). Wu et al.^(13)^ studied 48 cases of active TB in KTRs and found the following on CT: nodule or speckle, in 33 cases (68.8%); focal proliferation, in 20 (41.7%); pleural effusion, in 20 (31.3%); hilar or mediastinal lymph node enlargement, in 10 (20.8%); a miliary pattern, in 8 (16.7%); cavitation, in 5 (10.4%); and consolidation, in 3 (6.25%). Those authors did not specify the meaning of “focal proliferation”. In the present study, we performed a descriptive analysis because the number of cases in which there was a confirmed bacteriological diagnosis was small. In the Wu et al. study^(13)^, 22 (45.8%) of the 48 patients evaluated were diagnosed on the basis of clinical findings, without bacteriological confirmation. Despite our small sample size, we compared our findings with those of Pereira et al.^(12)^, Wu et al.^(13)^, and Gandhi et al.^(15)^, as detailed in Table 3. In patients treated with drugs that have stronger immunosuppressive effects, a miliary pattern is more common.

**Table 3:** Comparison among the radiological findings of Pereira et al.^(12)^, Wu et al.^(13)^, Gandhi et al.^(15)^, and the present study, in KTRs with pulmonary TB.

Imaging finding	Pereira et al.^(12)^ (N = 40)	Wu et al.^(13)^ (N = 48)	Gandhi etal.^(15)^ (N = 16)	Present study (N = 4)
Miliary nodules	16 (40.0%)	8 (16.7%)	3 (18.8%)	1 (25.0%)
Nodule or speckle^*^	-	33 (68.8%)	2 (12.5%)	-
Centrilobular nodules	9 (22.5%)	-	9 (56.2%)†	4 (100.0%)^†^
Focal proliferation	-	20 (41.7%)	-	-
Cavitation	9 (22.5%)	5 (10.4%)	7 (43.8%)	1 (25.0%)
Ground-glass opacity	6 (15.0%)	-	0 (0.0%)	1 (25.0%)
Consolidation	6 (15.0%)	3 (6.2%)	7 (43.8%)	3 (75.0%)
Lymphadenopathy	5 (12.5%)	10 (20.8%)	4 (25.0%)	1 (25.0%)
Pleural effusion	4 (10.0%)	15 (31.3%)	1 (6.2%)	0 (0.0%)

* Not a term recommended by the Fleischner Society^(14)^.

† With a tree-in-bud pattern.

Pre-transplant chest X-ray cannot rule out latent TB infection unless there are visible foci^(16)^, nor can it exclude pulmonary infections of other etiologies^(9)^. Among the 42 cases evaluated by Chen et al.^(16)^, chest X-ray revealed lung foci of pulmonary TB in 32 (76.2%); in eight cases (19.0%), chest X-ray reports only suggested pneumonia-like changes and did not effectively confirm the diagnosis of TB. Although the authors used CT to confirm the diagnosis in 14 patients (33.3%), they did not report the CT findings. In another study of KTRs with pulmonary infection, conducted by Mangalgi et al.^(9)^, the CT findings were not found to correlate with the infectious agent.

Gulati et al.^(8)^ concluded that HRCT provides more information than does chest X-ray in pulmonary infections, particularly in patients with TB. In one of the patients in our sample (patient 4), the chest X-ray was normal and HRCT revealed features consistent with active pulmonary TB. In patients 1 and 3, HRCT added information complementary to the chest X-ray findings, providing a more accurate diagnosis of pulmonary TB. Thus, our findings confirm the superiority of HRCT over chest X-ray in the diagnosis of active pulmonary TB in transplant recipients. Our results also corroborate the statement made by Wu et al.^(13)^: that the use of X-ray alone can delay the diagnosis of TB.

Although the clinical presentations are quite different, one of the main differential diagnoses of pulmonary TB in daily clinical practice is community-acquired pneumonia, a disease that is often treated with quinolones, which are second-line drugs for TB treatment. Inadvertent treatment with quinolones in patients with TB can delay the diagnosis and specific treatment of the disease, as well as raising concerns regarding the selection of resistant mycobacteria resulting from the use of monotherapy^(17,18)^.

In a study of 21 patients with latent TB infection who underwent chest X-ray and chest CT prior to lung transplantation^(19)^, lesions consistent with past TB were observed on the chest X-rays of only two patients (9.5%) and on the chest CT scans of 15 (71.4%). CT can also differentiate between active and inactive TB. Soft tissue infiltration, nodules, a miliary pattern, a tree-in-bud pattern, consolidation, and cavitation suggest active disease, whereas calcified nodules, bronchiectasis, and linear opacities suggest inactive disease.

Comparing the different patterns of the presentation of lung infections, Jiang et al.^(7)^ concluded that a tree-in-bud pattern is a statistically significant marker that differentiates between pulmonary TB and a bacterial lung infection. In our study, as in the literature^(15)^, HRCT provided key data to diagnose active pulmonary TB, based essentially on the finding of a tree-in-bud pattern. This significant marker to differentiate between active pulmonary TB and other lung infections is not visible on chest X-rays. Taking into account the variety of differential diagnoses for active pulmonary TB in KTRs and the fact that a delay in the specific diagnosis and treatment of this mycobacteriosis can increase the morbidity associated with the infection considerably, we believe that HRCT is always indicated when there is clinical suspicion of active pulmonary TB in an individual who has undergone KTX. There is clear evidence that CT is more sensitive and specific than is chest X-ray for the identification of TB activity, as well as facilitating the evaluation of the associated findings and complications.

Our study has some limitations. Only a small proportion of the patients assessed were found to have active pulmonary TB. Although the proportion was comparable to that reported in the literature and was therefore expected, the small sample size is a limiting factor. In our initial sample, there were two other patients who presented clinical and HRCT findings indicative of active pulmonary TB, as well as responding to the specific treatment. However, those patients could not be included in the analysis because mycobacteria were not detected in any of the clinical specimens. That could be explained by the fact that the laboratory tests used (smear microscopy and culture) have limited sensitivity^(20,21)^.Those cases seem to support the hypothesis that HRCT is highly accurate in the diagnosis of active pulmonary TB. Another potential limitation is that there are only a few studies on this topic. However, the sum of the experience obtained in this study and those reported in literature allows some conclusions to be reached. There is a need for studies with larger patient samples, preferably prospective studies, in order to better characterize the imaging patterns in this specific group of patients, as well as to determine whether or not there are differences between KTRs and the general population regarding the findings of pulmonary TB.

In the present study, HRCT provided more data than did chest X-ray, highlighting the tree-in-bud pattern in all of our patients. In cases of suspected active pulmonary TB in KTRs, HRCT should always be performed because of its significant superiority over chest X-ray for this diagnosis, as well as its greater sensitivity in relation to sputum tests. However, due to the high cost and limited availability of HRCT, especially in lowand middle-income countries, we do not advocate its use for TB detection in KTRs who are asymptomatic. In such cases, we recommend the routine use of chest X-ray in three views (apical-lordotic, anteroposterior, and lateral). However, under any pre-transplant or, especially, post-transplant clinical suspicion of infection, HRCT has proven to be far superior to chest X-ray, and it should always be used for early detection, in the differential diagnosis, and at the immediate beginning of the treatment for TB in KTRs.

## CONCLUSION

Over a 3.5-year period of follow-up period after KTX, the proportion of KTRs diagnosed with active pulmonary TB was 0.52%, which is consistent with data in the literature. The fact that HRCT provided greater data than did chest X-ray, revealing a tree-in-bud pattern in 100% of our patients, indicates that chest HRCT is superior to chest X-ray for confirming a diagnosis of active pulmonary TB. We therefore recommend that HRCT always be performed in cases of suspected active pulmonary TB in KTRs.
